# Cladribine treatment in pediatric-onset multiple sclerosis: real-world clinical outcomes and safety insights

**DOI:** 10.3389/fimmu.2026.1727925

**Published:** 2026-05-01

**Authors:** Esther Ganelin-Cohen, Yair Wexler, Sraya Kraus, Ayal Rozenberg

**Affiliations:** 1 Gray Faculty of Medical and Health Sciences, Tel Aviv University, Tel Aviv, Israel; 2 Neuroimmunological Clinic, Institute of Pediatric Neurology, Schneider Children’s Medical Center of Israel, Petah Tikva, Israel; 3 The George S Wise Faculty of Life Sciences, Tel Aviv University, Tel Aviv-Yafo, Israel; 4 The Ruth and Bruce Rappaport Faculty of Medicine, Technion Israel Institute of Technology, Haifa, Israel; 5 Department of Neurology, Rambam Health Care Campus, Haifa, Israel

**Keywords:** cladribine, disease modifying therapies (DMTs), NEDA, pediatric-onset multiple sclerosis (POMS), relapsing remitting multiple sclerosis (RRMS)

## Abstract

**Introduction:**

Pediatric-onset multiple sclerosis (POMS) poses unique therapeutic challenges due to high inflammatory activity, early cognitive and psychosocial impact, and difficulties with long-term treatment adherence. This retrospective cohort study evaluated the real-world safety and clinical outcomes of cladribine tablets compared with standard disease-modifying therapies (DMTs) in children and adolescents with POMS.

**Methods:**

A retrospective cohort of 47 POMS patients followed at two tertiary neuroimmunology centers was included: 31 received cladribine and 16 other DMTs. Inclusion required at least two years of continuous therapy and annual MRI monitoring. The control group comprised patients who remained on standard DMTs due to stable or well-tolerated disease, while exclusion criteria included prior use of high-potency biological therapies, specifically natalizumab or anti-CD20 agents. Clinical and radiological outcomes were analyzed using standard statistical tests and mixed-effects models.

**Results:**

Mean age at onset was 15.1 ± 2.5 years in the cladribine group and 13.8 ± 2.2 years in controls. Baseline disease duration was longer among cladribine-treated patients (3.8 ± 3.4 vs. 2.3 ± 1.8 years). NEDA rates were comparable between groups at Year 1. Relapse rates evolved differently over time (Group × Year interaction p = 0.028), reflecting differences in longitudinal patterns rather than a direct comparison of treatment efficacy. MRI and OCT measures showed no significant differences between groups. Withdrawal rates were lower with cladribine (9.4% vs. 52.6%), and no severe adverse events occurred.

**Conclusion:**

Cladribine demonstrated favorable safety, high adherence and comparable outcomes to standard DMTs, supporting its feasibility as an off-label option for selected pediatric patients.

## Introduction

Neuroinflammatory disorders in childhood pose unique challenges due to their potential impact on brain development, cognitive maturation, and immune regulation. Pediatric Onset Multiple Sclerosis (POMS) is a prototypical example, characterized by a highly inflammatory disease course and early cognitive and psychosocial consequences compared with adult-onset MS (AOMS) )^1^–^3^). Coping with chronic neuroinflammatory disease during a critical developmental period imposes a substantial physical and emotional burden on affected children and their families.

Despite significant therapeutic advances in adult MS, the number of disease-modifying therapies (DMTs) formally approved for pediatric use remains limited. Fingolimod was the first oral agent approved by the U.S. Food and Drug Administration (FDA) for pediatric relapsing MS, and additional agents, including dimethyl fumarate and teriflunomide, have since received pediatric approval in specific jurisdictions (4). Consequently, clinicians often rely on off-label administration of adult-approved DMTs based on real-world experience and safety extrapolation. Treatment adherence in adolescents is another major challenge, as irregular medication intake and needle aversion are common and contribute to breakthrough disease activity and treatment failure.

Cladribine is a synthetic purine analog that acts as an immune reconstitution therapy, selectively depleting lymphocytes while sparing innate immune function (5). Its unique dosing regimen—only 8–10 treatment days per year over two consecutive years-provides durable suppression of autoimmune activity, potentially improving adherence in younger patients who struggle with daily or injectable therapies (6).

In addition, weight-based dosing of cladribine (7) allows precise adjustment for pediatric patients, in contrast to many DMTs whose fixed adult doses require empirical modification or pose safety concerns in low-weight individuals. Clinical trials in adults have demonstrated favorable efficacy and safety profiles, with benefits often outweighing the risks and without evidence of increased malignancy compared with the general population (8–14). These findings are particularly reassuring when considering long-term safety in pediatric patients who face decades of exposure risk.

Conducting clinical trials in POMS remains difficult due to its low prevalence, heterogeneity, and ethical limitations, resulting in a paucity of evidence regarding the use of cladribine in this age group. Real-world studies are therefore essential to provide early data on feasibility and safety.

Based on its pharmacologic mechanism, established safety in adults, and adherence advantages, this study aimed to evaluate the safety, tolerability, and efficacy of cladribine tablets compared with standard DMTs in pediatric-onset MS patients.

## Materials and methods

### Study design and population

This retrospective cohort study included POMS patients followed at tertiary pediatric neuroimmunology clinics. Eligible participants were aged ≤18 years at disease onset and fulfilled the 2017 McDonald criteria for multiple sclerosis.

Inclusion criteria were: (1) continuous treatment with a single disease-modifying therapy (DMT) for at least two years; (2)availability of annual MRI follow-up; and (3) complete clinical and laboratory documentation.

Exclusion criteria included prior treatment with high-potency biological therapies (natalizumab or anti-CD20 agents); incomplete medical records; and follow-up shorter than 24 months.

### Treatment groups

Thirty-one patients received cladribine tablets and sixteen received other DMTs, including interferon-β, glatiramer acetate, dimethyl fumarate, and diroximel fumarate.

All eligible cladribine-treated patients during the study period were included consecutively. Eligibility for the control cohort required availability of complete annual MRI data and documentation of ≥24 months of single disease-modifying therapy (DMT).

The control group consisted of POMS patients who remained on standard DMTs without escalation to high-efficacy therapy during the study period. Controls were comparable to cladribine-treated patients in age and sex at baseline.

### Off-label treatment policy and ethical approval

Off-label administration of DMTs approved for adults was carried out in accordance with institutional and national regulatory standards.

The study protocol was reviewed and approved by the Institutional Review Boards (Helsinki Committees) of all participating centers.

Use of cladribine in pediatric patients, which falls outside the approved age indication, was permitted through a formal regulatory mechanism that authorizes the medical use of drugs outside their labeled indications or age limits upon submission of a justified physician request.

This process requires institutional endorsement and approval by the relevant national health authority prior to dispensing the medication through the healthcare provider.

### Data collection and safety monitoring

Clinical data were collected from regular follow-up visits of patients who maintained continuous treatment with a specific DMT for at least two years.

Inclusion required patients to have undergone annual MRI scans as part of their routine monitoring.

Relapses were defined as new neurological deficits attributable to demyelinating activity and were counted from treatment start; annual relapse outcomes refer to relapses occurring within each 12-month follow-up interval.

We examined safety outcomes, including any side effects associated with initiating a new DMT, as well as treatment adherence and compliance throughout the study period.

Radiological measures included T2 and gadolinium-enhancing lesion counts on brain MRI. MRI metrics are reported as lesion counts (number of lesions) and/or as the proportion of patients with ≥1 new/enlarging T2 lesion or ≥1 Gd+ lesion, as specified.

Optical coherence tomography (OCT) parameters, including retinal nerve fiber layer (RNFL) and ganglion cell layer (GCL) thickness, were analyzed when available.

NEDA (No Evidence of Disease Activity) was defined according to the standard NEDA-3 criteria as the absence of clinical relapses, absence of confirmed disability progression (EDSS increase ≥ 1.0 point sustained for ≥ 6 months), and no new or enlarging T2 or gadolinium-enhancing lesions on brain MRI during follow-up (15).

### Statistical analysis

Several longitudinal outcomes were compared between cladribine-treated patients and controls over two years of follow-up, including No Evidence for Disease Activity (NEDA) rate, relapse rate, imaging activity (T2 and contrast-enhancing lesions), and Optical Coherence Tomography (OCT) measurements of the retinal nerve fiber layer (RNFL) and of the ganglion cell layer (GCL) thickness. Binary outcomes were analyzed using mixed-effects logistic regression models with treatment group, follow-up year, and their interaction as the primary fixed effects and patient ID as a random intercept. The square-root transformed number of baseline contrast-enhancing lesions and number of baseline T2 lesions were retained as fixed effects in the final models following model selection using likelihood ratio tests. Additional covariates considered but not retained included sex, MS age of onset, number of relapses at baseline, EDSS at baseline, disease duration and treatment line (number of prior treatment changes). RNFL and GCL thickness were analyzed using linear mixed models with the same primary effect structure. Eye (left/right) and the number of baseline contrast-enhancing lesions were retained following model selection. Study withdrawal rate was analyzed using logistic regression with treatment group and baseline contrast-enhancing lesions as fixed effects. A mixed-effects logistic regression model with year as a fixed effect and patient ID as a random intercept was used to analyze the occurrence of lymphocytes nadir grade 3 and above.

Significance of fixed effects in the mixed models was assessed using likelihood ratio tests, and in the logistic regression model using type II Wald’s test. Unless otherwise specified, reported probabilities and means represent estimated marginal means derived from the fitted models. Unless otherwise specified, demographic and baseline characteristics were compared between treatment groups using t-tests and Fisher’s exact tests. P-values under 0.05 were considered statistically significant.

Analysis was conducted in R, utilizing the lme4 (16), car (17) and emmeans (18) packages.

## Results

### Demographic and baseline characteristics

A total of 47 POMS patients were included: 31 treated with cladribine and 16 receiving other DMTs. Baseline demographic, clinical data and distribution of DMT agents are summarized in **Table 1**.

**Table 1:** Baseline demographic and clinical characteristics of the study population.

Variable		Entire sample n = 47	Treatment group		p-value
			Cladribine n = 31	Control n = 16	
Sex	Female	30 (64%)	21 (68%)	9 (56%)	0.53
	Male	17 (36%)	10 (32%)	7 (44%)
MS age of onset (years)	Mean ± SD Median (Q1,Q3)	14.6 ± 2.5 15.0 (13.0,16.3)	15.1 ± 2.5 15.0 (13.3,17.0)	13.8 ± 2.2 14.3 (12.1,15.5)	0.08
Disease duration at baseline (years)	Mean ± SD Median (Q1,Q3)	3.3 ± 3.0 2.5 (1.0,4.8)	3.8 ± 3.4 3.0 (1.0,5.5)	2.3 ± 1.8 1.8 (0.9,3.0)	0.04
Brain Number of lesions at baseline	T2	12.9 ± 9.0 10.0 (6.0,16.0)	13.2 ± 8.7 14.0 (6.0,16.0)	12.5 ± 9.8 9.0 (6.8,13.2)	0.82
	Enhanced	0.54 ± 0.84 0 (0,1)	0.60 ± 0.91 0 (0,1)	0.44 ± 0.73 0 (0,1)	0.53
ON/Cervical spine Lesions at baseline	T2	1.2 ± 1.6 0.0 (0.0,2.0)	1.4 ± 1.8 1.0 (0.0,3.0)	0.7 ± 1.1 0.0 (0.0,1.0)	0.09
	Enhanced	0.17 ± 0.52 0 (0,0)	0.16 ± 0.58 0 (0,0)	0.19 ± 0.40 0 (0,0)	0.86
EDSS at baseline	Mean ± SD Median (Q1,Q3)	1.03 ± 0.63 1.00 (1.00,1.25)	1.10 ± 0.69 1.00 (1.00,1.75)	0.91 ± 0.49 1.00 (0.88,1.00)	0.28
Number of relapses at baseline	Mean ± SD Median (Q1,Q3)	0.72 ± 0.90 1 (0,1)	0.74 ± 1.03 0 (0,1)	0.69 ± 0.60 1 (0,1)	0.63
NEDA at baseline		13 (26%)	9 (29%)	4 (25%)	1
DMT	Cladribine Interferon-β Glatiramer acetate Dimethyl fumarate Diroximel		31 (100%)	6 (38%) 1 (6%) 8 (50%) 1 (6%)	
Treatment lines (Cladribine group)	First line (naïve) Second line Third line Fourth line			11 (35%) 7 (23%) 12 (39%) 1 (3%)	

Values are presented as mean ± SD or median (Q1–Q3) for continuous variables and as n (%) for categorical variables.

Comparisons between Cladribine and Control groups were made using t-tests for continuous variables and Fisher’s exact test for categorical variables. MS, multiple sclerosis; NEDA, No Evidence of Disease Activity; ON, optic neuritis; DMT, Disease-modifying therapy.

The mean age at MS onset was 15.1 ± 2.5 years in the cladribine group and 13.8 ± 2.2 years among controls (p = 0.08). Baseline disease duration was longer in cladribine treated patients (3.8 ± 3.4 vs 2.3 ± 1.8 years, p = 0.04).

Sex distribution and baseline Expanded Disability Status Scale (EDSS) scores were comparable between groups.

### Clinical outcomes (NEDA and relapses)

**Table 2** summarizes the p-values and odds ratios for the clinical outcomes and MRI outcomes, and **Table 3** summarizes the estimated rates over the follow-up period.

**Table 2:** Fixed effect p-values and odds ratios obtained from mixed-effect logistic regression results for clinical and MRI measures.

Measure	Fixed effect	p-value	Year 1 OR (95% CI)	Year 2 OR (95% CI)
NEDA	Group	0.47	1.33 (0.53,3.31)	1.01 (0.21,4.98)
	Year	0.0037 **		
	Group x Year	0.63		
Relapses	Group	0.41	0.92 (0.29,2.98)	3.77 (0.48,29.59)
	Year	< 0.001 ***		
	Group x Year	0.028 *		
New T2 brain lesions	Group	0.24	0.38 (0.04,3.27)	0.97 (0.08,11.79)
	Year	0.0079 **		
	Group x Year	0.59		
Contrast-enhancing lesions	Group	0.25	2.17 (0.60,7.88)	2.95 (0.27,32.73)
	Year	0.020 *		
	Group x Year	0.71		

Odds ratios (OR) and their confidence intervals were derived from the mixed-effect logistic regression model coefficients. Asterisks represent p-values, where “*” is under 0.05, “**” is under 0.01, and “***” is under 0.001.

**Table 3:** Percentage of patients exhibiting NEDA, relapses and imaging changes in each treatment year.

Measure	Group	Baseline (95% CI)	Year 1 (95% CI)	Year 2 (95% CI)
NEDA	Cladribine	27% (14,46)	42% (28,57) 35% (21,52)	59% (31,83)
	Control	17% (7,38)		59% (33,81)
Relapses	Cladribine	38% (21,59)	27% (16,42) 29% (13,51)	18% (7,39)
	Control	73% (46,90)		6% (0.9,27)
New T2 brain lesions	Cladribine	56% (34,76)	14% (5,33) 32% (12,62)	23% (6,59)
	Control	57% (30,81)		46% (20,74)
Contrast-enhancing lesions	Cladribine	39% (20,62)	23% (12,38)	12% (3,36)
	Control	29% (9,61)	12% (4,29)	4% (0.6,26)

Percentages and their 95% confidence intervals were obtained from the mixed-effects logistic regression models using estimated marginal means.

**Figure 1:**
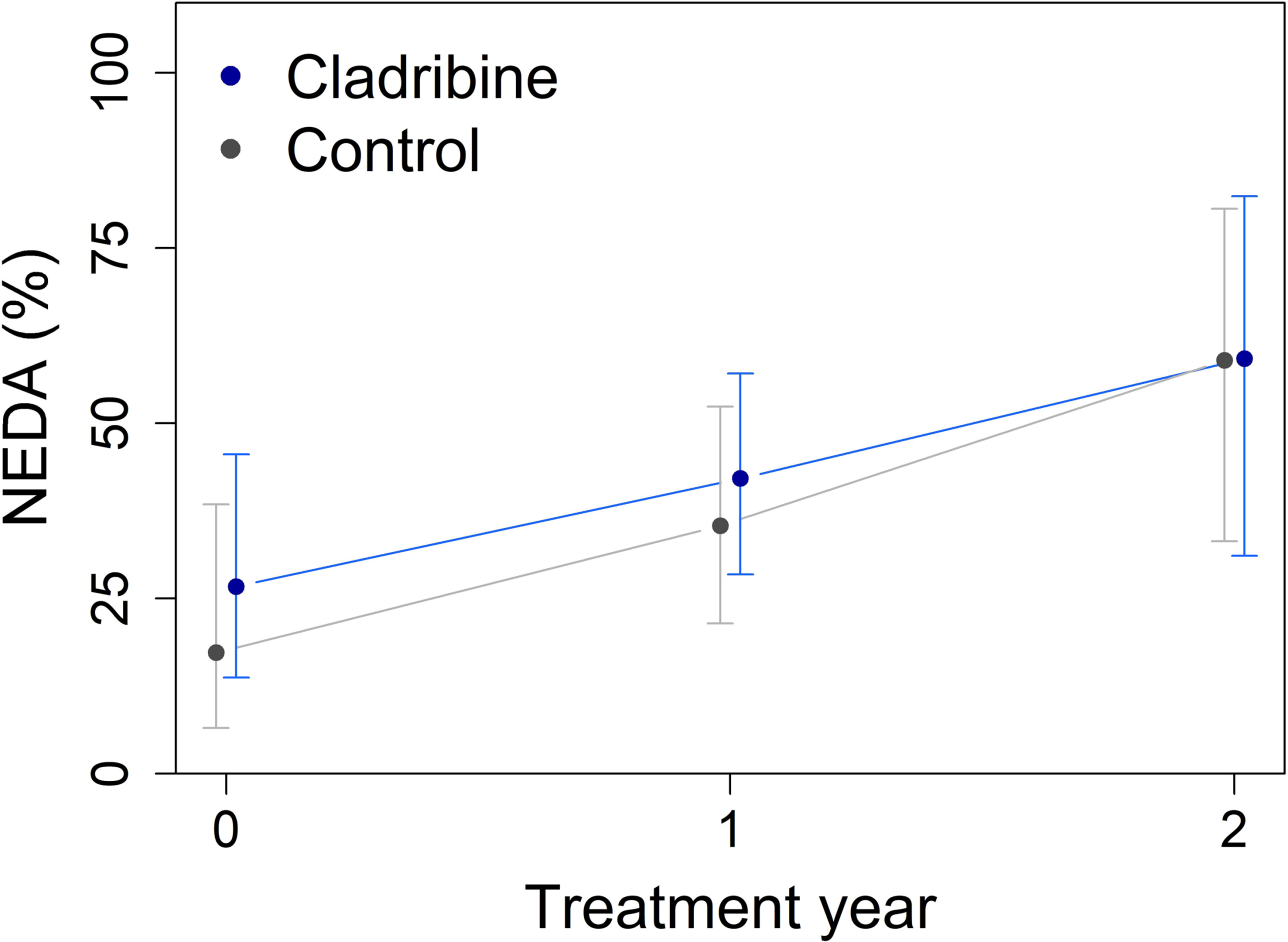
Percentage of patients achieving no evidence of disease activity (NEDA) at baseline, year 1, and year 2 in the cladribine (N = 31) and control groups (N = 16), obtained from mixed-effect logistic regression using estimated marginal means. Error bars indicate 95% confidence intervals. No significant differences were observed between groups at any time point.

Over the follow-up period, the proportion of patients achieving NEDA was similar in both groups (**Figure 1**). Mixed-effect logistic regression revealed no significant group effect (p = 0.47) or Group × Year interaction (p = 0.63). Likewise, no significant differences were found between groups in consecutive years in the rates of NEDA improvement (achieving NEDA after failing to do so) or deterioration (losing NEDA after prior achievement) (**Table 4**).

**Table 4:** Percentage of patients whose diagnosis improved or deteriorated as compared to the previous year.

Year	Group	Improved (95% CI)	Deteriorated (95% CI)
1	Cladribine	34% (15,59)	44% (14,79)
	Control	39% (15,70)	50% (7,93)
2	Cladribine	38% (8,81)	9% (0.2,41)
	Control	45% (14,81)	25% (3,65)

For improved, only patients who had not achieved NEDA the previous year were considered. Percentages represent estimated marginal means derived from the mixed-effects logistic regression. For *deteriorated* only patients who had achieved NEDA the previous year were considered. Due to very low number of candidates, no logistic model was fit. The values represent the raw percentages, and confidence intervals were derived using the Clopper-Pearson method.

**Figure 2:**
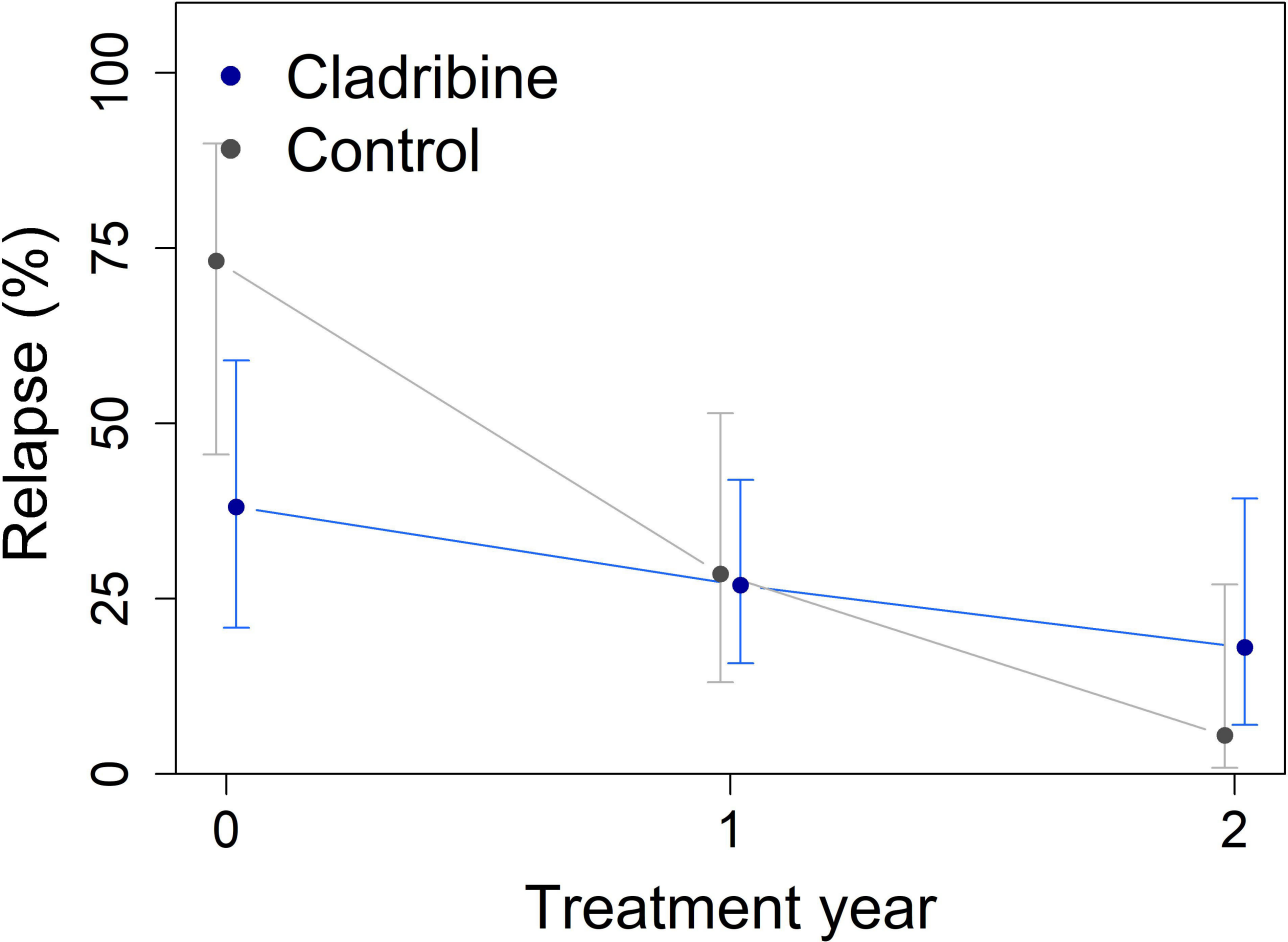
Annualized relapse rates in the cladribine (N = 31) versus control groups (N = 16) across two years of follow-up, obtained from mixed-effect logistic regression using estimated marginal means. The Group × Year interaction effect was significant (p = 0.028), reflecting differences in longitudinal relapse patterns between groups. Error bars indicate 95% confidence intervals.

The relapse rate differed between groups (Group × Year interaction p = 0.028, **Figure 2**), reflecting differences in longitudinal patterns rather than a direct comparison of treatment efficacy. No sustained disability progression was observed in either group during the study period.

### MRI findings

**Figure 3:**
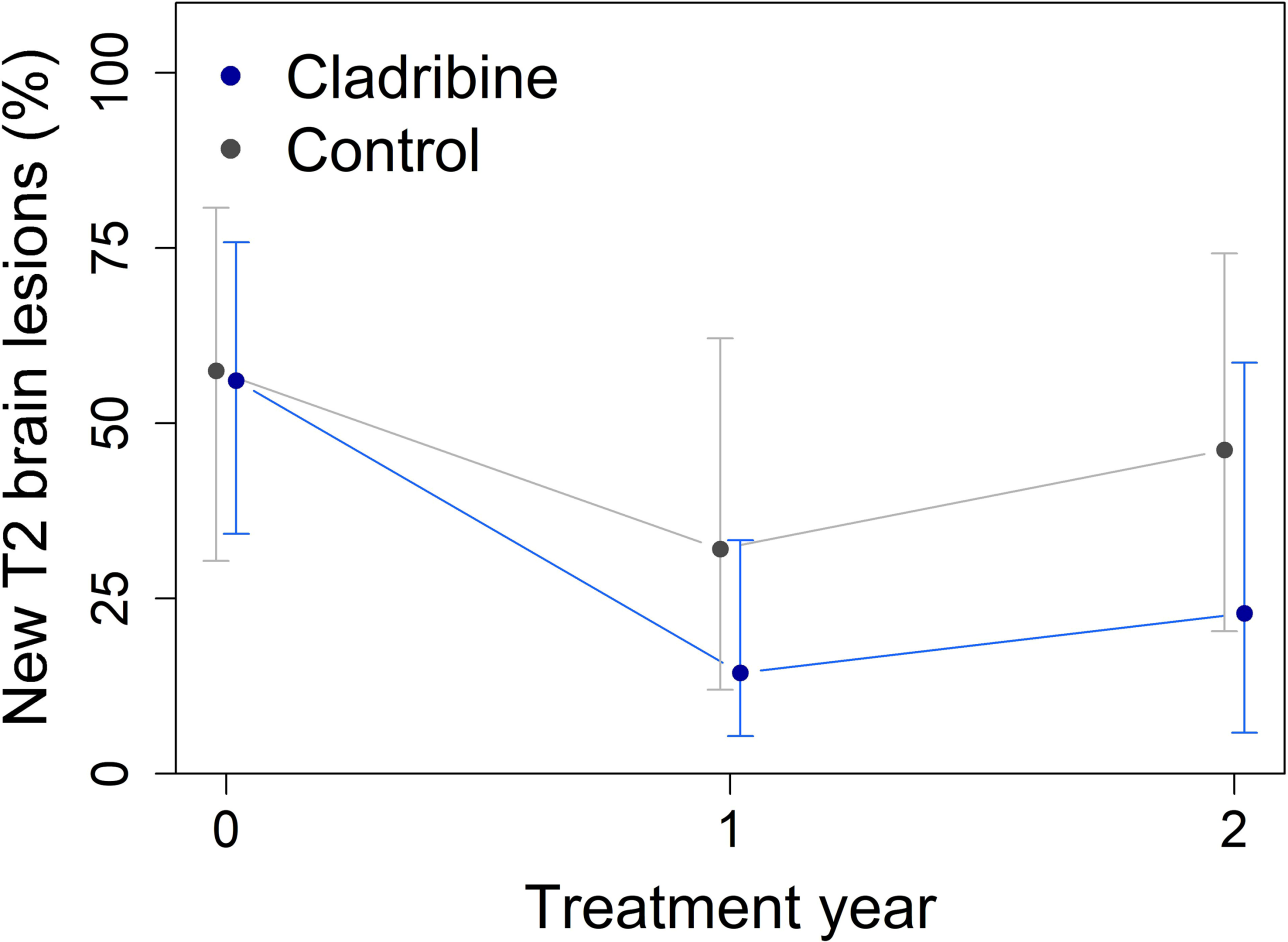
Percentage of patients with new T2 brain lesions over two years of follow-up in both groups, the cladribine (N = 31) and the control groups (N = 16) obtained from mixed-effect logistic regression using estimated marginal means. The proportion was reduced in two years by 33% in the cladribine group and by 11% in the controls, without a statistically significant difference. Error bars indicate 95% confidence intervals.

**Figure 4:**
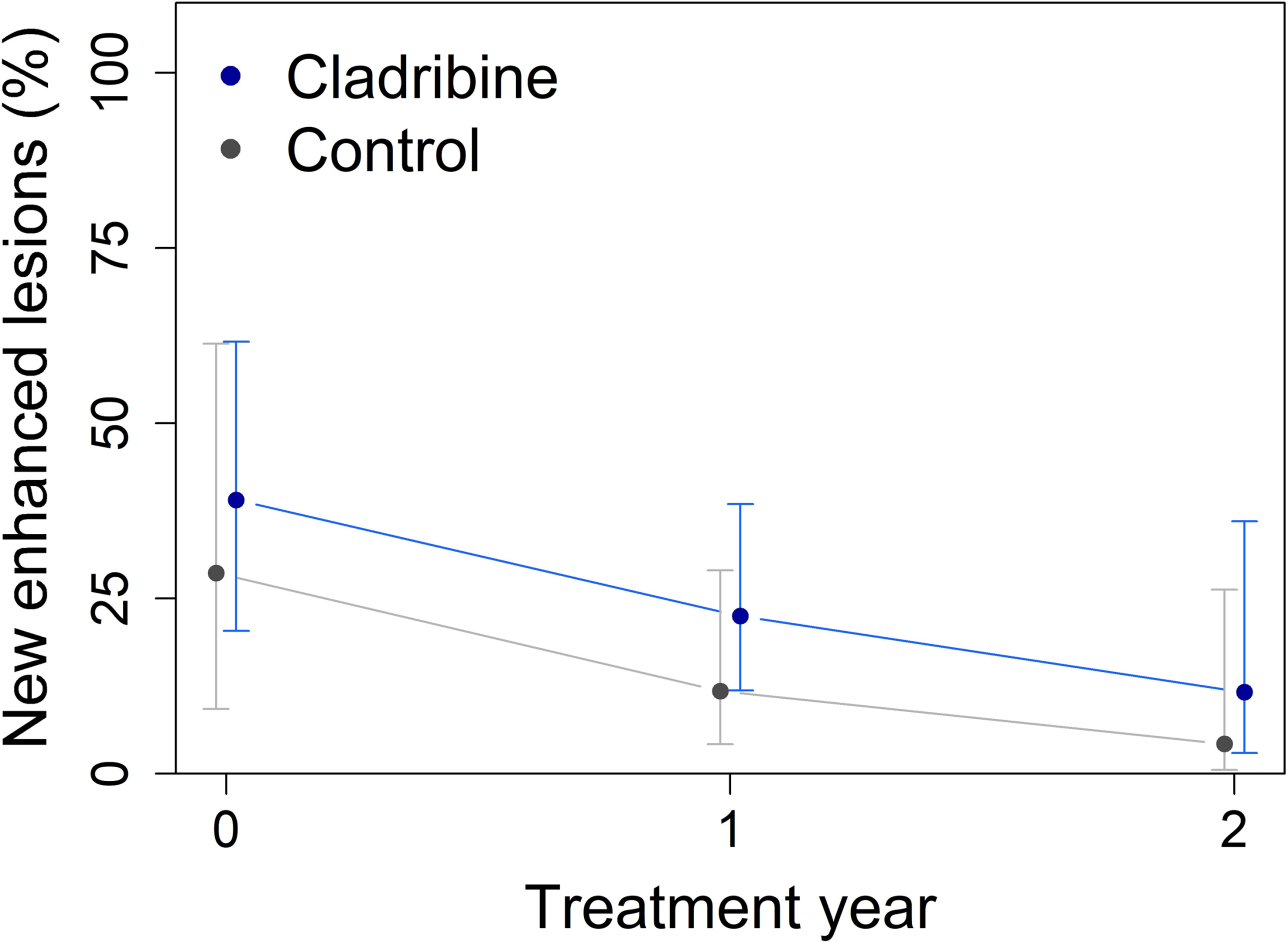
Rates of gadolinium-enhancing lesions on brain MRI at baseline and after two years, obtained from mixed-effect logistic regression using estimated marginal means. Enhancement declined steadily in both groups, the cladribine (N = 31) and the control groups (N = 16) with the cladribine group higher on average by 9% (p = 0.25). Error bars indicate 95% confidence intervals.

Both groups demonstrated a reduction in the proportion of patients with new or enlarging T2 lesions (Year effect, p = 0.0079) or gadolinium-enhancing lesions (p = 0.020) over the follow up period compared with the baseline, but no significant differences were observed between groups (**Tables 2**, **3**). Nevertheless, the proportion of patients with new T2 brain lesions numerically decreased over the two-year follow-up, by 33% in the cladribine group and by 11% in the control group (**Figure 3**). Enhancement rates declined comparably in both groups during follow-up; however, the enhancement in the cladribine group was on average 9% higher (p = 0.25, **Figure 4**).

### Optical coherence tomography findings

**Figure 5:**
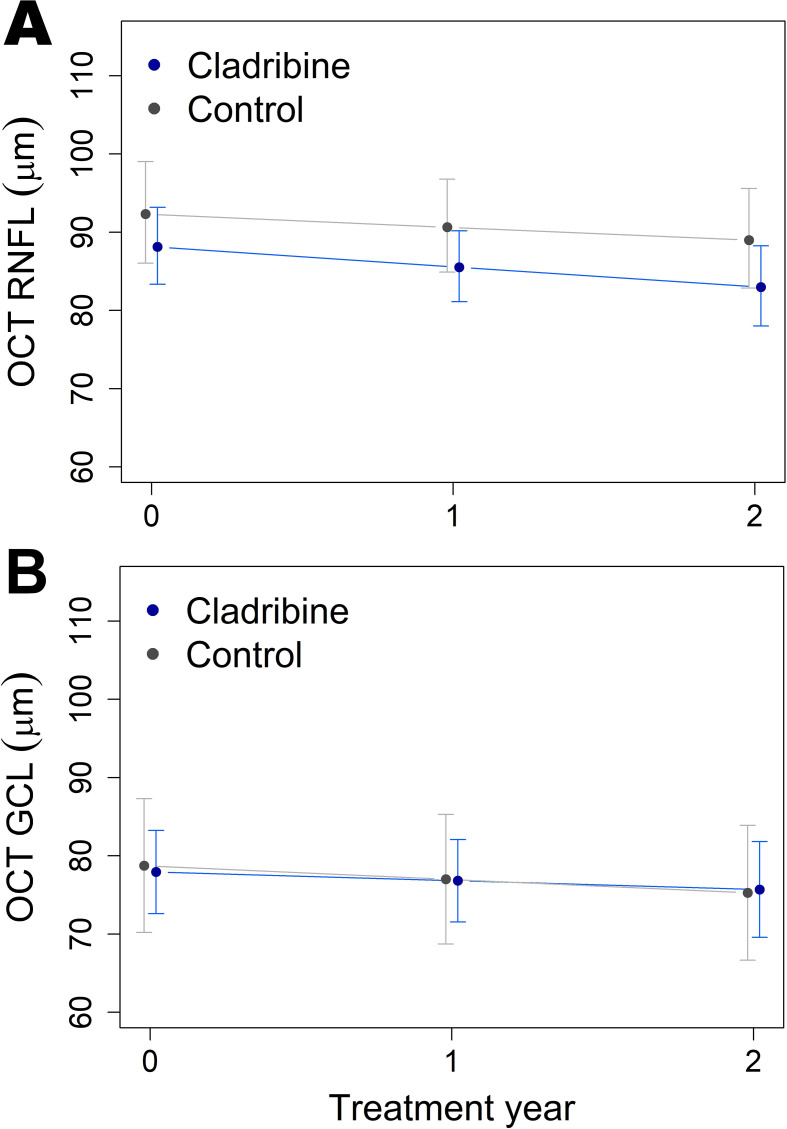
Optical coherence tomography (OCT) measures, reflecting changes in thickness of **(A)** retinal nerve fiber layer (RNFL) and **(B)** ganglion cell layer (GCL) over two years, obtained from linear mixed models using estimated marginal means. Both treatment groups, the cladribine (N = 31) and the control groups (N = 16) exhibited mild and comparable RNFL thinning (p = 0.012 Year effect). GCL thinning was not significant in either group. Error bars indicate 95% confidence intervals.

Over the two-year period, patients exhibited a significant gradual thinning of the retinal nerve fiber layer (RNFL, p = 0.012*, **Figure 5A**; **Table 5**), with no significant difference between groups. No significant thinning of the ganglion cell layer (GCL, p = 0.10) was observed in either group (**Figure 5B**; **Table 5**). Detailed OCT data are presented in **Table 5**, **Figure 5**.

**Table 5:** Optical coherence tomography (OCT) findings.

Tissue	Fixed effect	p-value	Group	OCT (µm) (95% CI)		
				Baseline	Year 1	Year 2
RNFL	Group	0.16	Cladribine Control	88 (83,93) 92 (86,99)	86 (81,90) 90 (85,97)	83 (78,88) 89 (83,96)
	Year	0.012 *				
	Group x Year	0.55				
GCL	Group	0.94	Cladribine Control	78 (72,83) 79 (70,87)	77 (72,82) 77 (69,85)	76 (70,82) 75 (67,84)
	Year	0.10				
	Group x Year	0.71				

Results of the linear mixed-models comparing OCT measurements between the groups. OCT measurements (in µm) and their 95% confidence intervals were obtained from the models using estimated marginal means. Asterisks represent p-values, where “*” is under 0.05, “**” is under 0.01, and “***” is under 0.001. Abbreviations: OCT, Optical Coherence Tomography; RNFL, Retinal nerve fiber layer; GCL, Ganglion cell layer.

### Safety and treatment adherence

**Figure 6:**
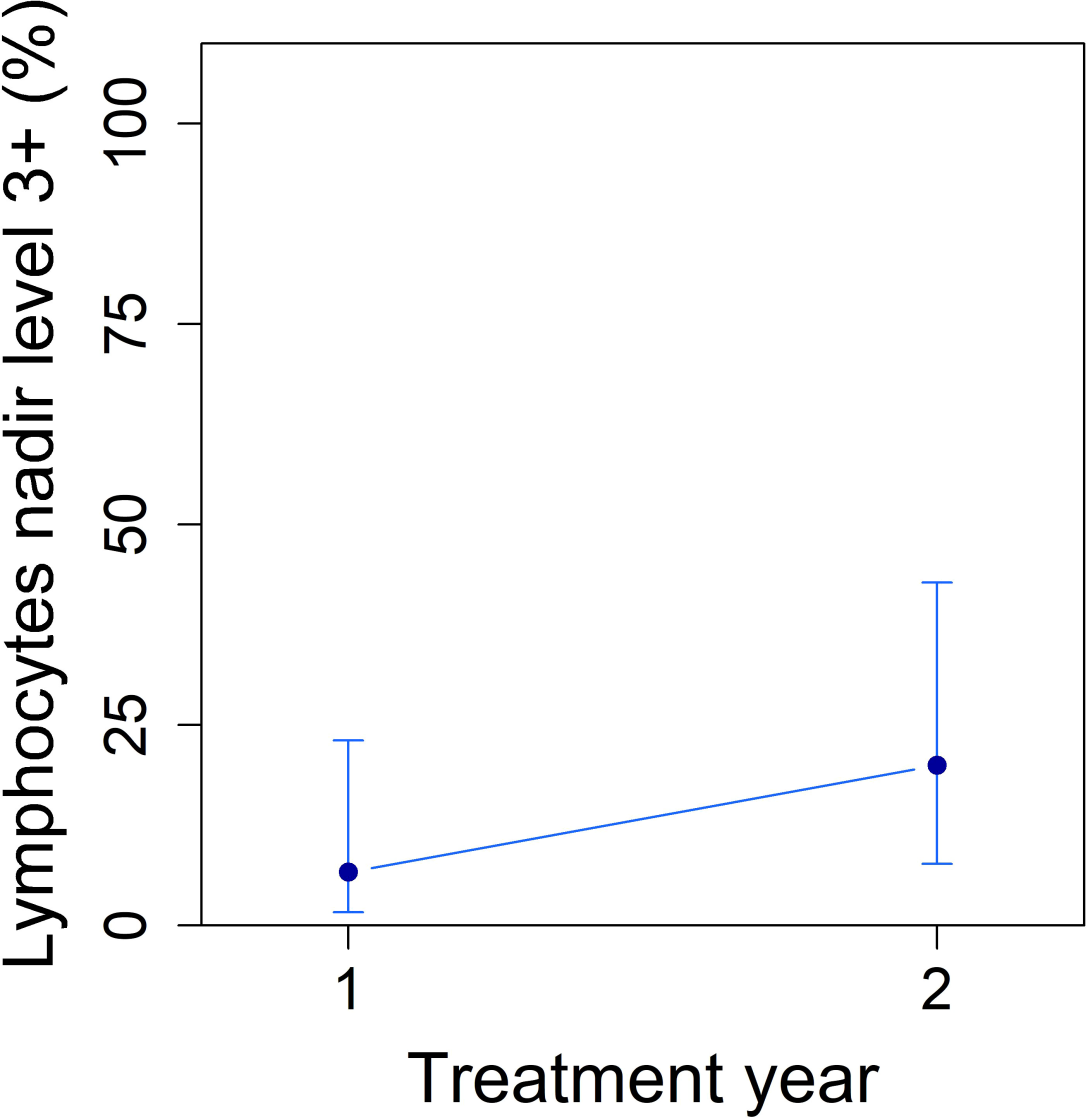
Lymphocyte counts trends in the cladribine group (N = 31) during follow-up, obtained from mixed-effect logistic regression using estimated marginal means. Grade 3 lymphopenia was observed in 20% of patients, resolving spontaneously without treatment interruption. Error bars indicate 95% confidence intervals.

Cladribine treatment was well tolerated. No severe or unexpected adverse events were reported in the cladribine group. The most notable laboratory abnormality was grade 3 lymphopenia, observed in 20% of patients (**Figure 6**), which resolved spontaneously without requiring treatment interruption or additional interventions. Fatigue (reported in 16% of patients) was the most common clinical complaint and was self-limited. No hematologic, hepatic, or infectious complications necessitating hospitalization were recorded.

Importantly, no patients discontinued cladribine due to adverse events.

**Figure 7:**
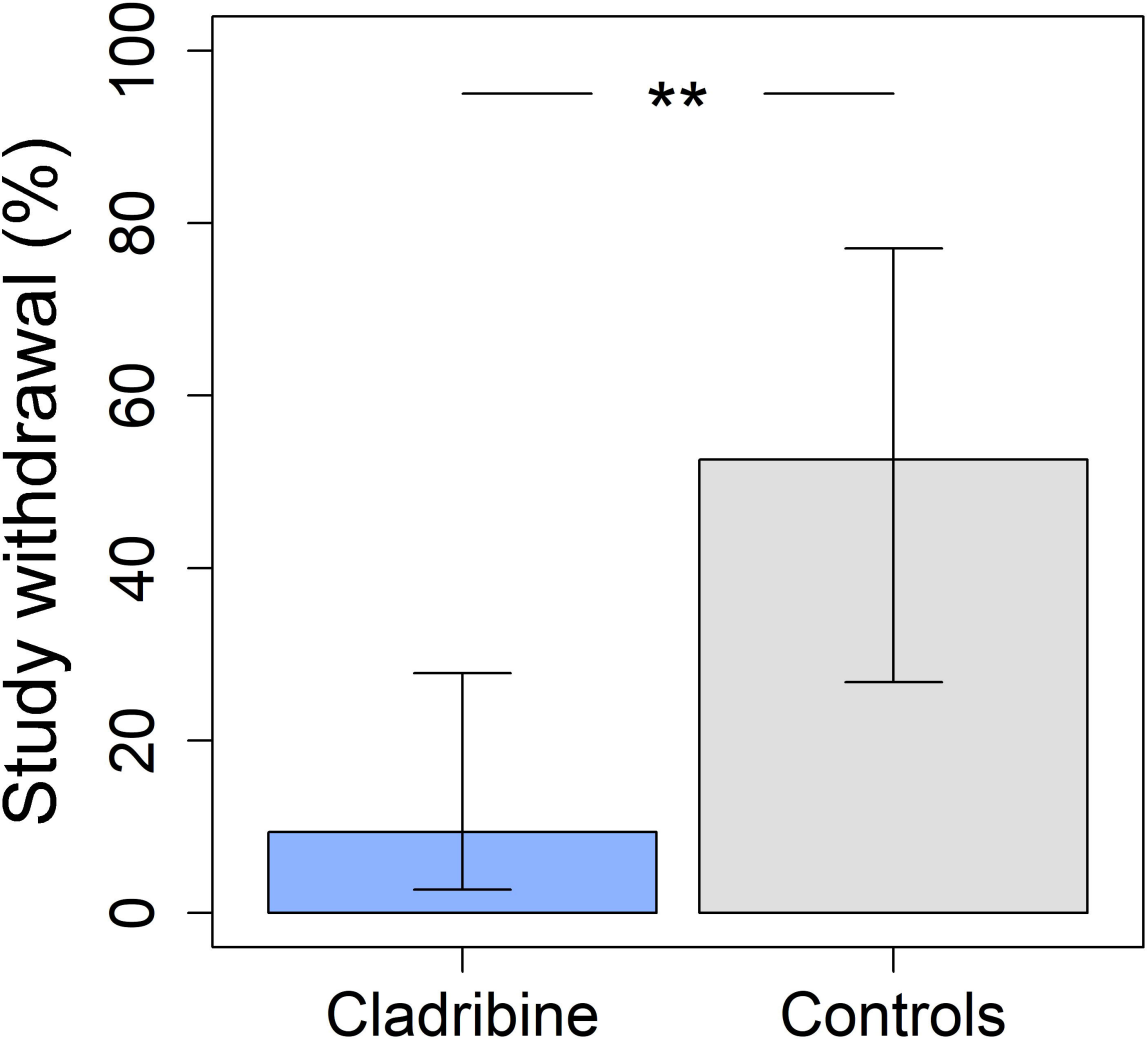
Treatment adherence and discontinuation rates in the cladribine (N = 31) and control groups (N = 16), obtained from a logistic regression model using estimated marginal means. The cladribine group had a significantly lower withdrawal rate (9.4% vs. 52.6%, p = 0.0047 **). “**” represents p-value under 0.01 but over 0.001. Error bars indicate 95% confidence intervals.

Treatment adherence was significantly higher among cladribine-treated patients, reflected by a lower discontinuation rate of 9.4% at Year 2 compared to 52.6% in the controls (p = 0.0047, **Figure 7**), with an odds ratio of 0.094 (95% CI: 0.02 – 0.59). All discontinuations in the control group were related to poor tolerance or lack of efficacy.

## Discussion

In this real-world retrospective study, we evaluated clinical outcomes, radiological outcomes, safety, and treatment adherence of cladribine compared with standard DMTs in POMS. Despite its off-label use, cladribine demonstrated comparable control of disease activity, an encouraging safety profile, and superior adherence—even among patients who presented with more active disease at baseline.

At treatment initiation, patients in the cladribine group had a longer disease duration and a higher proportion of gadolinium-enhancing lesions, suggesting that clinicians preferentially tended to reserve cladribine for patients with more active or treatment-refractory disease.

This baseline imbalance reflects confounding by indication, an inherent limitation of retrospective studies, and provides essential context for interpreting the observed differences in relapse patterns over the follow-up period.

The observed difference in relapse trajectories should be interpreted with caution. Although the Group × Year interaction remained statistically significant after adjustment for baseline differences, this finding reflects variation in longitudinal patterns within a real-world cohort and does not support a direct comparison of treatment efficacy. Accordingly, this study is not designed to establish superiority or inferiority of cladribine relative to other therapies, but rather to provide real-world data on its safety, tolerability, and feasibility in pediatric-onset multiple sclerosis.

The relapse activity likely reflects the underlying disease severity rather than a genuine treatment failure. Supporting this interpretation, MRI outcomes showed comparable reductions in new T2 and enhancing lesions across both groups, indicating effective suppression of inflammatory activity once cladribine was initiated. Beyond confounding by indication, the delayed onset of cladribine’s immune reconstitution effect may also contribute to early disease activity. Clinical stabilization may require 12–18 months, and inflammatory events during the first year may occur before maximal benefit is achieved.

Similarly, NEDA rates did not differ significantly between groups. Mixed-effects modeling confirmed the absence of group or interaction effects, and the wide confidence intervals underline the limited power of this small cohort. Nonetheless, maintaining NEDA in roughly one-third of cladribine-treated patients—despite greater baseline disease activity—represents a clinically relevant outcome, consistent with findings from adult real-world data.

When compared with adult clinical trial data, our results are broadly aligned with those of the CLARITY program (7, 19). In CLARITY, about 45–47% of adults-maintained NEDA after two years of cladribine treatment, versus roughly one-third in our pediatric cohort. This lower proportion likely reflects the greater baseline disease activity and treatment refractoriness of our real-world population. Importantly, the safety profile observed—limited to transient grade 3 lymphopenia without serious adverse events—closely parallels adult experience, supporting the biological consistency of cladribine’s immune-reconstitution effect across age groups.

The OCT findings complement these radiological observations by offering an additional structural perspective. Over the two-year follow-up, both treatment groups demonstrated gradual thinning of the retinal nerve fiber layer (RNFL) and ganglion cell layer (GCL)—a well-recognized manifestation of subclinical neuroaxonal loss in pediatric multiple sclerosis, even in the absence of optic neuritis (20–23). Previous pediatric studies have consistently shown that RNFL and GCL thinning is common in POMS and correlates with disease duration and inflammatory activity, serving as a reliable biomarker of cumulative neuronal injury (20, 24). The absence of significant between-group differences in our cohort suggests that cladribine does not accelerate neuroaxonal degeneration and may stabilize retinal integrity similarly to standard DMTs.

A particularly notable observation in this cohort was the superior treatment adherence among cladribine recipients. After adjusting for baseline differences, only 9.4% discontinued treatment compared with 52.6% in the control group (p = 0.0047). This difference is both statistically and clinically meaningful, especially in adolescents, where adherence challenges are a key barrier to long-term disease control. The simplified, short-course oral dosing schedule of cladribine likely contributed to this advantage and may represent a pragmatic therapeutic option in real-world pediatric settings. Importantly, none of the cladribine-treated patients discontinued due to adverse events, while nearly half of the control-group withdrawals were attributed to side effects, further reinforcing its tolerability profile.

The safety findings were consistent with those reported in adult cohorts. The most notable laboratory abnormality was grade 3 lymphopenia, observed in approximately 20% of cladribine-treated patients, which resolved spontaneously without intervention or treatment discontinuation. No severe infections or serious adverse events were observed. Follow-up beyond the primary two-year analysis was available for a subset of cladribine-treated patients (8 at Year 3 and 4 at Year 4). No new safety signals or unexpected adverse events were observed during this extended follow-up (data not shown).

This pattern supports the accumulating evidence that cladribine can be safely administered in carefully monitored pediatric populations.

In addition, while the current analysis focuses on the two-year active treatment period of cladribine, longer-term follow-up is essential to better characterize the durability of its effect. In particular, evaluation of NEDA3 status at Year 4 and beyond may help define sustained disease control and identify baseline characteristics associated with long-term response.

Beyond short-term safety and efficacy, the question of long-term management following completion of cladribine therapy warrants consideration. Given the limited long-term evidence for immune reconstitution therapies in pediatric multiple sclerosis, post-treatment management after completion of the 2-year cladribine regimen requires structured monitoring and individualized decision-making. Continued follow-up should include regular clinical assessments and at least annual brain MRI. Disease reactivation—defined by clinical relapse and/or new MRI activity—should prompt reassessment of therapeutic strategy, guided by disease severity, prior treatment exposure, and safety considerations. Given the paucity of pediatric data, such decisions should be undertaken in specialized centers and incorporate shared decision-making with patients and their families.

Nonetheless, these findings should be interpreted in light of several methodological limitations. The retrospective, non-randomized design introduces selection bias and residual confounding. Prior treatment exposure and relapse history could have influenced both treatment allocation and outcomes. The Cladribine group had longer disease duration, greater prior treatment exposure, and higher baseline MRI inflammatory activity. Although these factors were accounted for in the statistical models, residual confounding cannot be excluded.

In addition, the small sample size limits power and precision, as reflected by the wide confidence intervals. MRI and OCT follow-ups were clinically driven rather than standardized, and OCT data were incomplete for several participants.

An additional limitation is the lack of systematic volumetric MRI measures, including assessment of thalamic atrophy, which is increasingly recognized as a sensitive marker of neurodegeneration in pediatric-onset multiple sclerosis. The retrospective design and variability in imaging protocols did not allow reliable longitudinal volumetric analysis. Future prospective studies incorporating standardized volumetric MRI measures may provide further insight into the neuroprotective effects of cladribine.

Despite these constraints, our findings provide valuable real-world insight into the use of cladribine in pediatric MS. The comparable efficacy, high adherence, and favorable safety profile observed in this cohort suggest that cladribine may offer a practical and acceptable treatment option for selected pediatric patients, particularly those who struggle with adherence to continuous injectable or oral DMTs.

In the evolving therapeutic landscape of pediatric-onset multiple sclerosis, treatment strategies increasingly include high-efficacy agents such as anti-CD20 therapies alongside immune reconstitution approaches. Cladribine may represent a useful option in selected patients, particularly when long-term adherence to continuous therapies is challenging or when a pulsed treatment approach is preferred.

Its short-course dosing regimen and favorable tolerability in this cohort support its role as a feasible alternative in specific clinical scenarios. However, its positioning relative to other high-efficacy therapies remains to be defined and requires further evaluation in prospective studies with longer follow-up. Given the gradual onset of effect of cladribine, the timing and patient selection for its use may warrant further consideration, particularly in relation to baseline disease activity.

## Conclusion

In conclusion, cladribine demonstrated a reassuring balance between efficacy, safety, and adherence in pediatric MS, even among patients with higher baseline disease activity. These findings support its potential role as an off-label immune reconstitution approach in adolescents with relapsing disease, pending confirmation in larger prospective, controlled studies.

## Data Availability

The original contributions presented in the study are included in the article/supplementary material. Further inquiries can be directed to the corresponding author.
